# The complete chloroplast genome of *Phlomoides betonicoides* (Lamiaceae), a traditional Tibetan medicinal herb

**DOI:** 10.1080/23802359.2019.1696248

**Published:** 2019-12-09

**Authors:** Yue Zhao, Turginov Orzimat Turdimatovich, Chun-Lei Xiang

**Affiliations:** a CAS Key Laboratory for Plant Diversity and Biogeography of East Asia, Kunming Institute of Botany, Chinese Academy of Sciences, Kunming, China;; b University of Chinese Academy of Sciences, Beijing, China;; c Institute of Botany, Academy of Sciences Republic of Uzbekistan, Tashkent, Uzbekistan

**Keywords:** Complete plastomic sequences, Lamioideae, Phlomoides, tribe Phlomideae

## Abstract

The species *Phlomoides betonicoides* is used medicinally and mainly distributed in southwest China. The first complete plastid genome sequence of *P. betonicoides* reported here was 151,777 bp long, with the large single copy (LSC) region of 83,205 bp, the small single copy (SSC) region of 17,370 bp and two inverted repeats (IRa and IRb) of 25,601 bp. The plastome contained 114 genes, including 80 protein-coding genes, four ribosomal RNA genes, and 30 transfer RNA genes. The overall GC content was 38.5%. Result from phylogenetic analysis suggests that tribe Phlomideae (*Phlomoides*) is closely related to tribe Lamieae and tribe Leonureae in present study.

*Phlomoides* Moench, with about 150–170 species, is the largest genus of the tribe Phlomideae (Lamioideae, Lamiaceae) (Scheen et al. 2010; Bendiksby et al. 2011; Salmaki et al. 2012). The genus has undergone major species radiation in China, Central Asia, and the Iranian highland (Khosroshahi and Salmaki 2019). In China, some species have been used as Tibetan traditional medicine, such as *P. rotata* (Benth. ex Hook. f.) Mathiesen and *P. betonicoides* (Diels) Kamelin & Makhm.

Fresh leaves of *Phlomoides betonicoides* were collected from Lijiang, Yunnan, southwest China (100°17′8.56″E, 27°12′34.4″N). Voucher specimen (*C.L. Xiang 1289*) was deposited in the Herbarium of Kunming Institute of Botany, Chinese Academy of Sciences (KUN). Total genomic DNA was isolated using the CTAB method (Doyle and Doyle 1987) and sequenced on the Illumina HiSeq 2000 Sequencing System at BGI-Shenzhen (Shenzhen, Guangdong, China). The *de novo* assembling of the chloroplast genome was implemented in the GetOrganelle pipeline (https://github.com/Kinggerm/GetOrganelle, Jin et al. 2018), and the genome obtained was annotated using software Geneious v.11.0.3 (Kearse et al. 2012). The annotated plastid genome sequence has been deposited into the GenBank with the accession number MN617020.

The whole plastid genome of *P. betonicoides* was 151,777 bp in length, with a large single-copy (LSC) region (83,205 bp), a small single-copy (SSC) region (17,370 bp), and a pair of inverted repeats (IRa and IRb: 25,601 bp). The annotated genome comprised 114 genes, including 80 protein-coding genes, four ribosomal RNA genes (rrn16, rrn23, rrn4.5, rrn5), and 30 transfer RNA genes.

Eighteen genes were duplicated in the IR regions, including seven protein-coding genes (*ndhB*, *rpl2*, *rpl23*, *rps12*, *rps7*, *ycf2*, *ycf15*), four ribosomal RNA genes (rrn16, rrn23, rrn4.5, rrn5), and seven transfer RNA genes (trnA-UGC, trnI-CAU, trnI-GAU, trnL-CAA, trnN-GUU, trnR-ACG, trnV-GAC). The overall GC content of *P. betonicoides* plastid genome is 38.5% (LSC, 36.7%; SSC, 32.5%; IRs, 43.4%).

Maximum likelihood (ML) phylogenetic analyses were conducted using RAxML v8.1.11 (Stamatakis 2014) as implemented on the Cyber infrastructure for Phylogenetic Research (CIPRES) Science Gateway (http://www.phylo.org/, Miller et al. 2010), employing the GTR + G model with 1000 bootstrap iterations (-#|-N). Other parameters used the default settings. Phylogenetic analysis based on 80 protein-coding genes of 35 representative plastomes within the family Lamiaceae suggests that *Phlomoides* is a member of the subfamily Lamioideae (Figure 1) and sister to a clade comprising tribes Lamieae (*Lamium galeobdolon* L. and *L*. *album* L.) and Leonureae (*Leonurus japonicus* Houtt.).

**Figure 1.**
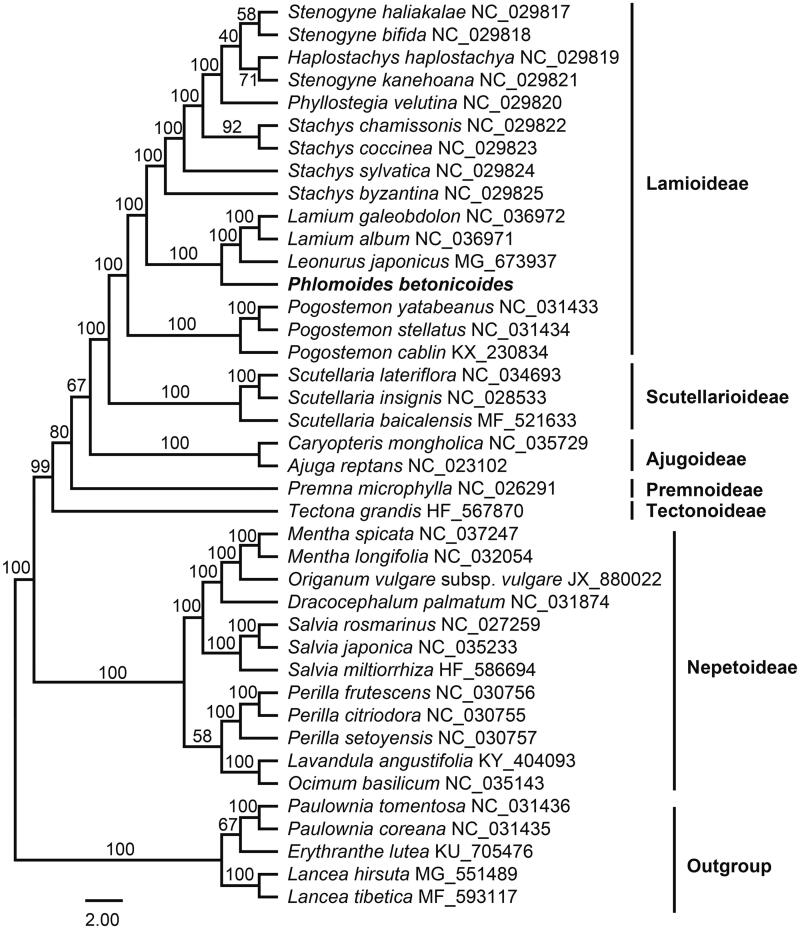
Maximum likelihood tree of Lamiaceae inferred from 80 protein-coding genes of 40 plastomes (include five outgroups). Bootstrap values are indicated above branches.
